# Comparative epidemiology of porcine circovirus type 3 in pigs with different clinical presentations

**DOI:** 10.1186/s12985-017-0892-4

**Published:** 2017-11-13

**Authors:** Shao-Lun Zhai, Xia Zhou, He Zhang, Ben M. Hause, Tao Lin, Runxia Liu, Qin-Ling Chen, Wen-Kang Wei, Dian-Hong Lv, Xiao-Hui Wen, Feng Li, Dan Wang

**Affiliations:** 1 Guangdong Key Laboratory of Animal Disease Prevention, Animal Disease Diagnostic Center, Institute of Animal Health, Guangdong Academy of Agricultural Sciences, Guangzhou, 510640 China; 2 Department of Biology and Microbiology, South Dakota State University, Brookings, SD 57007 USA; 3 Department of Veterinary and Biomedical Sciences, South Dakota State University, Brookings, SD 57007 USA; 4 College of Veterinary Medicine, South China Agricultural University, Guangzhou, 510642 China; 5 Cambridge Technologies, Worthington, MN 56187 USA; 6 Department of Chemistry and Biochemistry, South Dakota State University, Brookings, SD 57007 USA

**Keywords:** Porcine circovirus type 3, Comparative epidemiology, Asymptomatic, Diarrhea, Respiratory disease

## Abstract

**Background:**

Porcine circovirus type 3 (PCV3), as an emerging circovirus species, was reported to be widely circulating in the United States, China, South Korea and Poland. Previous studies revealed that PCV3 was mainly concentrated in sick animals with respiratory disease, skin disease, reproductive disorders and so on. However, the circulating status of PCV3 in pigs with other clinical presentations (especilly asymptomatic or diarrhea) was not well established.

**Findings:**

In this study, to conduct a comparative epidemiological survey of PCV3, 80 weaned pig serum samples with severe respiratory disease (SRD), 175 weaned pig serum samples with mild respiratory disease (MRD), 216 asymptomatic weaned pig serum samples, 35 diarrheal weaned pig samples and 35 non-diarrheal weaned pig samples were collected from eight provinces of China. Via qPCR testing, PCV3 was circulating in all sampling provinces, with total positive rates varying from 1.04% to 100%. Interestingly, the PCV3-positive rate was significantly higher in weaned pigs with SRD (63.75%, 51/80) than in those weaned pigs with MRD (13.14%, 23/175) and asymptomatic pigs (1.85%, 4/216) (*P* < 0.01). Similarly, the PCV3-positive rate was significantly higher in diarrheal weaned pigs (17.14%, 6/35) than in non-diarrheal weaned pigs (2.86%, 1/35) (*P* < 0.05). Moreover, the lower Ct values of qPCR were frequently found in those weaned pigs or fattening pigs with respiratory disease and diarrhea rather than that in asymptomatic pigs. Sequence analysis showed that low genetic diversity existed among those PCV3 sequences collected from pigs with different clinical presentations.

**Conclusions:**

The present study further extends evidence that newly described PCV3 widely circulates in six additional provinces of Southern and Northern China and has high similarity to previously reported isolates. As an emerging virus of swine, although the present case-control study reveals that PCV3 has a potential association with swine respiratory disease and diarrhea, further investigations into the pathogenesis are needed to ascertain the role of PCV3 in swine health.

**Electronic supplementary material:**

The online version of this article (10.1186/s12985-017-0892-4) contains supplementary material, which is available to authorized users.

## Background

At present, small non-enveloped DNA circoviruses of concern to animal and human health are classified in two genera (C*ircovirus* and *Cyclovirus*) in the *Circoviridae* family. The genus *Circovirus* consists of 27 species, while the genus *Cyclovirus* has 43 species [1]. Porcine circoviruses are members of the genus C*ircovirus* with two recognized species, porcine circovirus type 1 (PCV1) and porcine circovirus type 2 (PCV2). PCV1 can infect and replicate in swine, but is not known to cause any obvious disease [2]. While PCV2 infection can result in significant production and economic losses to the worldwide swine industry [3, 4]. Clinically, PCV2 can cause porcine circovirus disease (PCVD) or porcine circovirus-associated disease (PCVAD), which is characterized by postweaning multisystemic wasting syndrome (PMWS), porcine dermatitis and nephrotic syndrome (PDNS), interstitial pneumonia, and reproductive disorders [5, 6].

Recently, a novel circovirus significantly divergent from PCV1 and PCV2, provisionally designated porcine circovirus type 3 (PCV3), was identified in U.S. swine herds [7, 8]. The full-length genome sequence of newly found PCV3 was 2000 nucleotides, which was considerably larger than those of PCV1 and PCV2 (~1760 and 1780 nucleotides, respectively) [9]. Actually, prior to identification of PCV3, PCV3-like sequences were also reported in different farmed animals several years ago, which revealed possible early distribution of PCV3 in animal species [10, 11]. It is interesting that PCV3, similar to PCV2, has a potential association with multi-systemic disease, PDNS and reproductive failure in pigs [7, 8]. At present, PCV3 also has been reported in China, South Korea and Poland, accompanying with widely geographical distribution [12–17]. Despite good progress in PCV3 epidemiology, the circulating status of PCV3 in pigs with different clinical presentations (especilly asymptomatic or diarrhea) was not well established. Therefore, the present study aims to perform comparative epidemiology of PCV3 in pigs with different clinical presentations.

## Methods

**Fig. 1:**
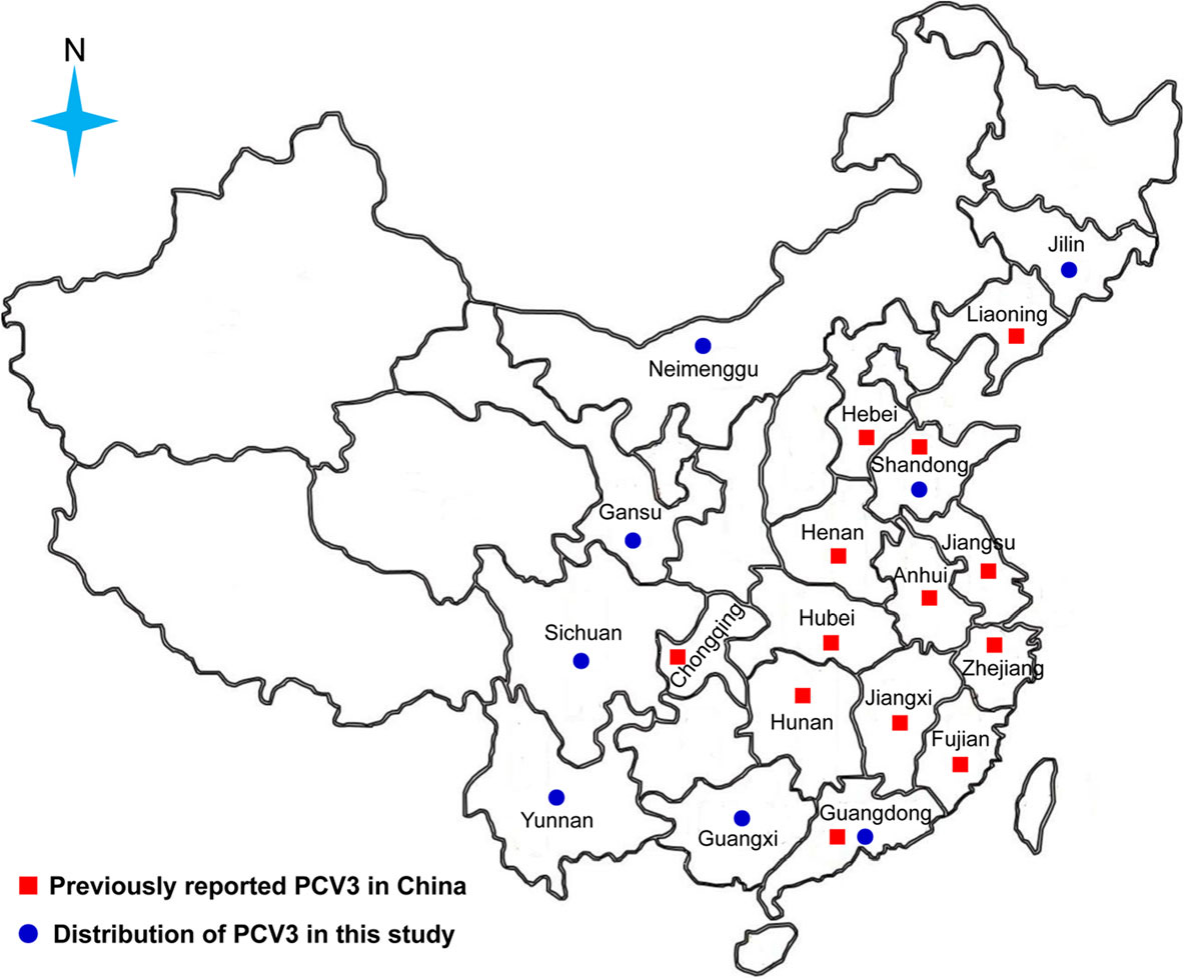
Geographical distribution of PCV3 in China

In 2016, two small-scale fattening pig farms (about 200 or 300 American Landrace pigs in total), locating in Guangdong province (southern China) and Jilin province (northern China) separated by more than 3, 000 km in distance (Fig. 1), suffered from severe respiratory disease (SRD). Pigs (2-3 months old) on both farms have received immunizations against PCV2, pseudorabies virus (PRV), classical swine fever virus (CSFV) and type 2 porcine reproductive and respiratory syndrome virus (PRRSV). However, the morbidity rate of pigs on the two farms was approximately 60%, the mortality in affected pigs was about 80%. From autopsy results of 18 individual dead pigs (*n* = 8 for farm A, *n* = 10 for farm B) (Additional file 1: Table S1), the similar macroscopic lesions were concentrated in the lungs and lymph nodes, which was characterized by PRRSV- or PCV2-like lesions including pulmonary interstitial widening, congestion and lymphadenopathy bleeding. These 18 pig lung samples were defined as the samples with SRD. To compare the detection results of PCV3 based on the same organ and same age of pigs, additional 80 swine (with SRD, 5-8 weeks old) serum samples, 216 asymptomatic weaned swine (5-8 weeks old) serum samples and 175 diseased weaned swine (5-8 weeks old) (with mild respiratory disease [MRD] including cough, softly panting, abdominal breathing) serum samples (Additional file 1: Table S1) were collected from Shandong province (*n* = 26), Gansu province (*n* = 120), Guangxi zhuang autonomous region (*n* = 34), Guangdong province (*n* = 130), Sichuan province (*n* = 65) and Yunnan province (*n* = 96) (Fig. 1). Moreover, 35 diarrheal weaned pig (6-7 weeks old) samples and 35 non-diarrheal weaned pig (6-7 weeks old) samples (Additional file 1: Table S1) were collected from Gansu province (*n* = 40) and Neimenggu autonomous region (*n* = 30) (Fig. 1). The above-mentioned samples were stored −80 °C until further use.

Prior to quantitative PCR (qPCR), 200 μL tissue supernatants or serum samples were used to perform viral DNA extraction according to the manufacturer’s instructions (TIANGEN Biotech Co., Ltd. Beijing, China). Viral DNA samples were stored at −80 °C until use. A previously established qPCR protocol was used, consisting of two specific primers (Forward, 5′–AGTGCTCCCCATTGAACG–3′; Reverse, 5′–ACACAGCCGTTACTTCAC–3′) and one modified probe (5′–FAM–ACCCCATGGCTCAACACATATGACC–BHQ1–3′) [8]. Moreover, the detection of PRRSV was performed according to a previous method [18]. The full-length genome sequence of PCV3 was amplified using the two pairs of overlapping PCR primers according to a previous description [13]. These two PCR fragments were cloned and sequenced. The corresponding sequencing results were spliced using SeqMan program (DNAStar software version 7). Sequence alignment and phylogenetic analysis based on three species of PCV sequences were performed using Clustal W program implemented in DNAStar software and MEGA 5.1 software, respectively. In addtion, qPCR detection results in pigs with different clinical presentations were analyzed by chisquare (χ^2^) test (Ziyue software), the value of *P* < 0.05 and *P* < 0.01 was considered significant and very significant, respectively.

## Results

**Fig. 2:**
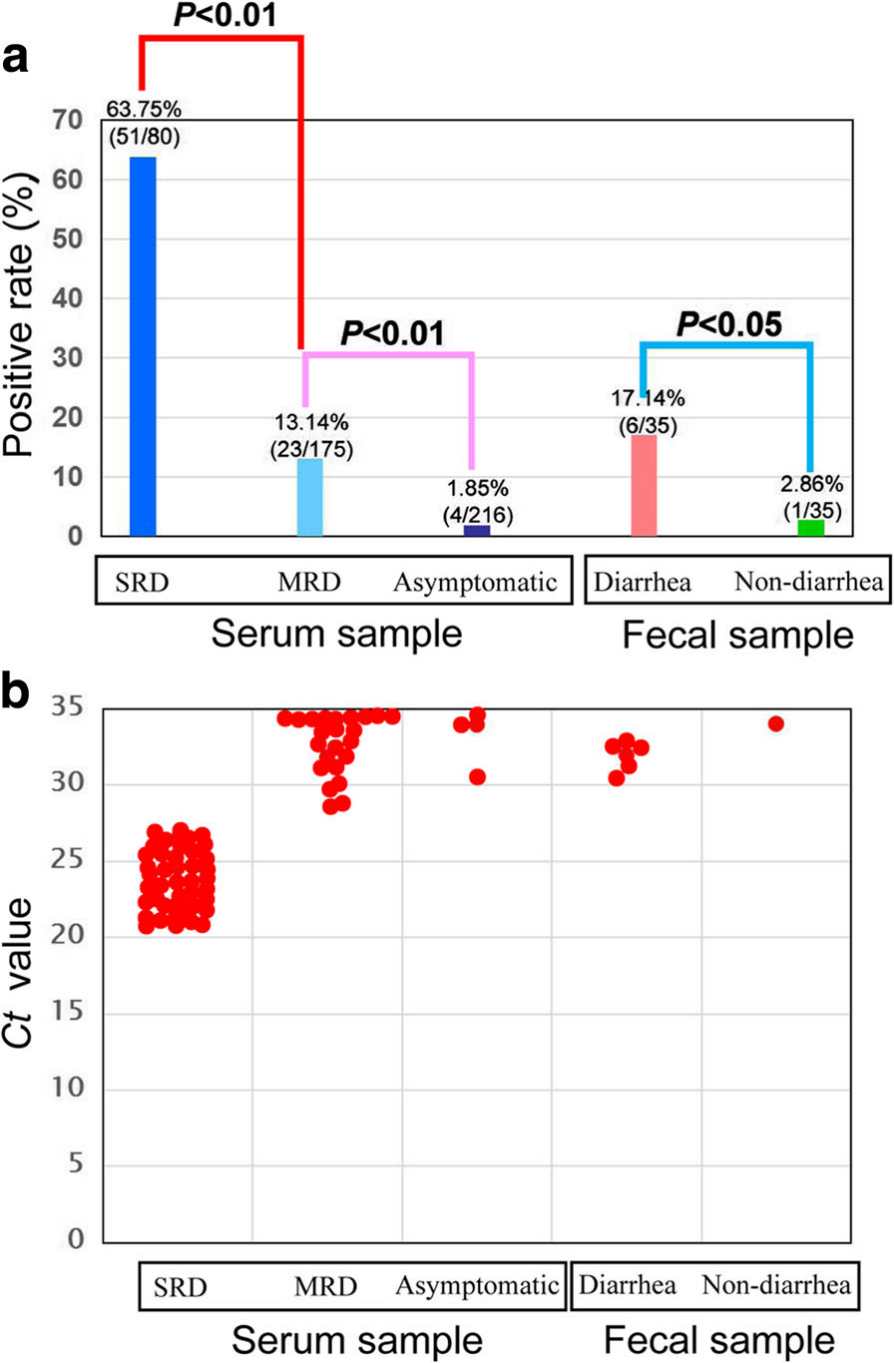
The detection results of PCV3 in pigs with different clinical presentations. **a** PCV3-positive rate in pigs with different clinical presentations; **b** Ct value of qPCR for the detection of PCV3 in pigs with different clinical presentations. Note: SRD means severe respiratory disease, while MRD means mild respiratory disease

After testing by qPCR, PCV3 was circulating in all sampling provinces, with total positive rates of 100% (8/8) in Jilin, 44.29% (62/140) in Guangdong, 7.69% (2/26) in Shandong, 3.75% (6/160) in Gansu, 8.82% (3/34) in Guangxi, 13.85% (9/65) in Sichuan, 1.04% (1/96) in Yunnan and 23.33% (7/30) in Neimenggu, respectively. Interestingly, the PCV3-positive rate was significantly higher in weaned pigs with SRD (63.75%, 51/80) than in those weaned pigs with MRD (13.14%, 23/175) and asymptomatic pigs (1.85%, 4/216) (*P* < 0.01) (Fig. 2a). Similarly, the PCV3-positive rate was significantly higher in diarrheal weaned pigs (17.14%, 6/35) than in non-diarrheal weaned pigs (2.86%, 1/35) (*P* < 0.05) (Fig. 2a). Moreover, the lower *Ct* values were frequently found in those samples collected from fattening or weaned pigs with respiratory disease and diarrhea rather than those from asymptomatic pigs (Fig. 2b). In addition, co-infection of PCV3 and PRRSV was found in 29 of 140 SRD pigs, not in MRD pigs and clinically asymptomatic pigs.

**Fig. 3:**
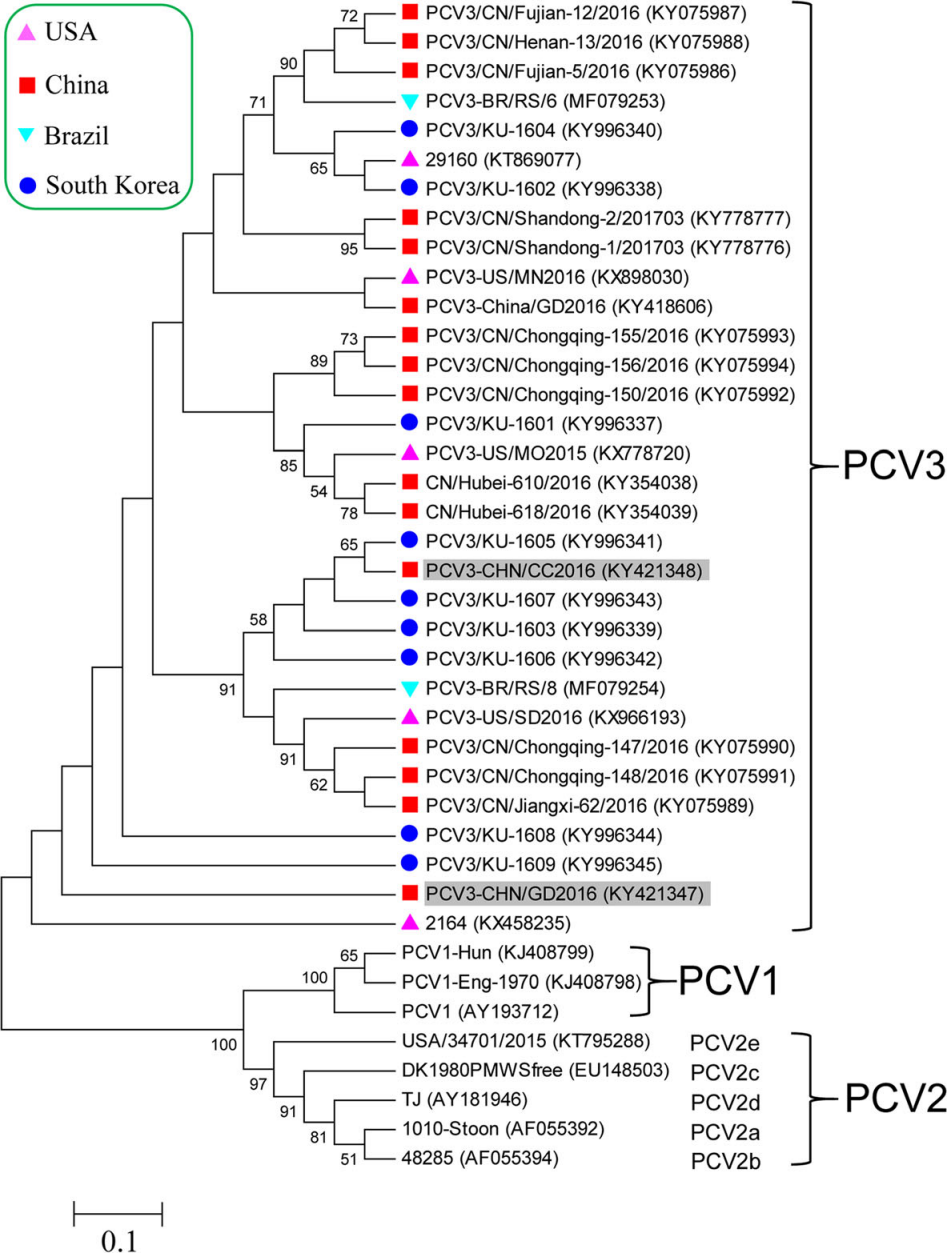
Phylogenetic analysis based on complete genome sequences of PCV1, PCV2 and PCV3. The phylogenetic tree was constructed by the Maximum Likelihood method using MEGA 5.1 software. One thousand bootstrap replicates (1000 times) were used to assess tree topology. Only bootstrap scores of at least 50 were retained. Scale bar indicates 10% nucleotide sequence divergence. The PCV3 isolates of PCV3-CHN/GD2016 and PCV3-CHN/CC2016 obtained in this study are indicated by *gray* shading

To further genetically characterize PCV3 in those positive samples with low Ct values (< 22), two representative genome sequences (PCV3-CHN/GD2016 and PCV3-CHN/CC2016) were obtained from south China and north China, respectively, and were deposited into GenBank database under accession numbers KY421347-KY421348. Like previously reported PCV3 sequences, their genomes were 2000 nucleotides in length. Multiple sequence alignment results showed that the two current PCV3 genome sequences shared > 97% nucleotide similarity with other known PCV3 sequences, revealing low levels of genetic variation among PCV3 isolates. Online Blastn alignment results showed that they mainly differed in ORF2 gene that encodes viral capsid protein (Data not shown). In addition, phylogenetic analysis showed that PCV3-CHN/GD2016 and PCV3-CHN/CC2016 clustered with those PCV3 isolates from the United States and Korea, and were distantly related to PCV1 and PCV2 (Fig. 3).

## Discussion

Previous studies suggested that PCV3 was common in PDNS cases and that it might play a role in reproductive failure and multi-systemic inflammation in U.S swine herds [7, 8]. Although there were other swine pathogens (including torque teno sus virus 1, TTSuV1; porcine hemagglutinating encephalomyelitis virus, PHEV; porcine astrovirus 4, PAstV4) present in PCV3-infected tissues, high numbers of PCV3 sequence reads by metagenomic sequencing suggested that PCV3 might play a potentially pathogenic role in those clinical cases [7, 8]. Similarly, immunohistochemistry localized PCV3 in typical PDNS lesions in the absence of PCV2 [8]. However, in four latest studies from China, single PCV3 infection and/or co-infection with PCV2 were reported in clinical cases [12, 13, 15, 16]. Surprisingly, during one of four studies, PCV3 had an abnormally high positive rate (85.7%, 12/14) in pig samples with productive failure, but PCV2, PRRSV and PRV were negative in these pigs [15]. In this study, we identified co-infection of PCV3 with PRRSV in SRD pigs, not in other pigs. Co-infection of pigs with PCV2 and PRRSV is known to exacerbate disease severity [19, 20]. A possible synergy between PCV3 and PRRSV or PCV2 co-infection with disease severity warrants further investigation. Additionally, low *Ct* values of PCV3 were frequently found in diseased pigs, not in asymptomatic pigs (Fig. 2b), which revealed that the high titers of PCV3, similar to PCV2, could contribute to the occurrence of clinical diseases in pigs [21]. Another interesting finding was that PCV3 was detected in fecal samples, which may suggest for a fecal-oral transmission by PCV3.

## Conclusions

To conclude, the present study further extends evidence that newly described PCV3 widely circulates in six additional provinces of Southern and Northern China and has high similarity to previously reported isolates. As an emerging virus of swine, although the present case-control study reveals that PCV3 has a potential association with swine respiratory disease and diarrhea, further investigations into the pathogenesis are needed to ascertain the role of PCV3 in swine health. Similar to PCV2 [22], the usage of viral infectious clone accompanying by clinical and histological methods could resolve this scientific issue.

## Additional file

Additional file 1: Table S1. The detection results of qPCR for PCV3 in collected samples. (DOC 65 kb)

## Abbreviations

CSFV: classical swine fever virus
MRD: mild respiratory disease
PAstV4: porcine astrovirus 4
PCV1: Porcine circovirus type 1
PCV2: Porcine circovirus type 2
PCV3: Porcine circovirus type 3
PCVAD: porcine circovirus-associated disease
PCVD: porcine circovirus disease
PDNS: porcine dermatitis and nephrotic syndrome
PHEV: porcine hemagglutinating encephalomyelitis virus
PMWS: postweaning multisystemic wasting syndrome
PRRSV: porcine reproductive and respiratory syndrome virus
PRV: pseudorabies virus
qPCR: quantitative PCR
SRD: severe respiratory disease
TTSuV1: torque teno sus virus 1
