# Supplementary material for: Comparative epidemiology of porcine circovirus type 3 in pigs with different clinical presentations
Source: Virol J. 2017 Nov 13;14:222. doi: 10.1186/s12985-017-0892-4 (PMC5683367; doi:10.1186/s12985-017-0892-4)
Supplement: Additional file 1: Table S1. — The detection results of qPCR for PCV3 in collected samples. (DOC 65 kb) [file 12985_2017_892_MOESM1_ESM.doc]

**Table S1 The detection results of qPCR for PCV3 in collected samples**

| Sample province | Farm | Sample type | Sample (*n*) | Positive rate | *Ct* value |
| --- | --- | --- | --- | --- | --- |
| Jilin | Farm A | Lung** | 8 | 100% (8/8) | 20.00, 20.98, 21.67, 22.62, 23.65, 35.00, 21.38, 22.55 |
| Guangdong | Farm B | Lung** | 10 | 50% (5/10) | 22.08, 20.99, 23.36, 21.22, 24.56 |
|  | Farm C | Serum* | 30 | 20% (6/30) | 31.81, 34.53, 34.53, 33.48, 34.38, 34.52 |
|  |  | Serum^†^ | 20 | 0% (0/20) | / |
|  | Farm D | Serum** | 20 | 60% (12/20) | 21.12, 23.87, 26,62, 25.32, 22,19, 23,21, 21.22, 23.54,  24.82, 26,54, 27.12, 20.28 |
|  | Farm E | Serum** | 20 | 65% (13/20) | 20.56, 21.69, 23,46, 21.63, 23,45, 22,35, 25.25, 22.39,  23.87, 22,38, 21.82, 23.78, 21.65 |
|  | Farm F | Serum** | 20 | 80% (16/20) | 23.59, 22.45, 22,76, 25.91, 24,09, 21,46, 23.27, 23.76,  26.54, 27,65, 25.85, 24.75, 22.56, 26,23, 24.89, 25.38 |
|  | Farm G | Serum** | 20 | 50% (10/20) | 20.56, 21.69, 23,46, 21.63, 23,45, 22,35, 25.25, 22.39,  23.87, 22,38 |
| Guangxi | Farm H | Serum* | 20 | 10% (2/20) | 32.14, 33.98 |
|  |  | Serum^†^ | 14 | 7.14% (1/14) | 33.94 |
| Gansu | Farm I | Serum* | 15 | 0% (0/15) | / |
|  |  | Serum | 15 | 0% (0/15) | / |
|  | Farm J | Serum* | 15 | 26.67% (4/15) | 29.93, 27.92, 31.64, 31.44 |
|  |  | Serum^†^ | 15 | 6.67% (1/15) | 33.91 |
|  | Farm K | Feces^§^ | 10 | 0% (0/10) | / |
|  |  | Feces^‡^ | 10 | 0% (0/10) | / |
|  | Farm L | Serum^†^ | 60 | 1.67% (1/60) | 30.41 |
|  | Farm M | Feces^§^ | 10 | 0% (0/10) | / |
|  |  | Feces^‡^ | 10 | 0% (0/10) | / |
| Shandong | Farm N | Serum* | 13 | 15.38% (2/13) | 28.93, 30.39 |
|  |  | Serum^†^ | 13 | 0% (0/13) | / |
| Sichuan | Farm O | Serum* | 35 | 22.86% (8/35) | 34.79, 34.43, 34.84, 34.59, 34.04, 34.61, 32.86, 31.98 |
|  |  | Serum^†^ | 30 | 3.33% (1/30) | 34.66 |
| Yunnan | Farm P | Serum* | 15 | 0% (0/15) | / |
|  |  | Serum^†^ | 15 | 0% (0/15) | / |
|  | Farm Q | Serum* | 17 | 0% (0/17) | / |
|  |  | Serum^†^ | 17 | 0% (0/17) | / |
|  | Farm R | Serum* | 15 | 6.67% (1/15) | 33.51 |
|  |  | Serum^†^ | 17 | 0% (0/17) | / |
| Neimenggu | Farm S | Feces^§^ | 15 | 40% (6/15) | 31.93, 31.15, 30.69, 32.80, 32.58, 32.89 |
|  |  | Feces^‡^ | 15 | 6.67% (1/15) | 34.00 |

** Those pigs had severe respiratory disease (SRD) including tachypnea, droopy appearance and nearly dying or dead. * Those pigs had mild respiratory disease (MRD) including cough, softly panting and abdominal breathing. ^†^ Asymptomatic. ^§^ Diarrhea. ^‡^ Non-diarrhea. / no *Ct* value.
